# Effectiveness of bereavement counselling through a community‐based organization: A naturalistic, controlled trial

**DOI:** 10.1002/cpp.2113

**Published:** 2017-08-29

**Authors:** Catherine Newsom, Henk Schut, Margaret S. Stroebe, Stewart Wilson, John Birrell, Mirjam Moerbeek, Maarten C. Eisma

**Affiliations:** ^1^ Department of Clinical Psychology Utrecht University Utrecht The Netherlands; ^2^ Department of Clinical Psychology and Experimental Psychopathology University of Groningen Groningen The Netherlands; ^3^ Cruse Bereavement Care Scotland United Kingdom; ^4^ Department of Methodology and Statistics Utrecht University Utrecht The Netherlands

**Keywords:** bereavement, complicated grief, counselling, grief, prolonged grief disorder

## Abstract

This controlled, longitudinal investigation tested the effectiveness of a bereavement counselling model for adults on reducing complicated grief (CG) symptoms. Participants (N = 344; 79% female; mean age: 49.3 years) were adult residents of Scotland who were bereaved of a close relation or partner, experiencing elevated levels of CG, and/or risks of developing CG. It was hypothesized that participants who received intervention would experience a greater decline in CG levels immediately following the intervention compared to the control participants, but the difference would diminish at follow‐up (due to relapse). Data were collected via postal questionnaire at 3 time points: baseline (T), post‐intervention (T + 12 months), and follow‐up (T + 18 months). CG, post‐traumatic stress, and general psychological distress were assessed at all time points. Multilevel analyses controlling for relevant covariates were conducted to examine group differences in symptom levels over time. A stepwise, serial gatekeeping procedure was used to correct for multiple hypothesis testing. A main finding was that, contrary to expectations, counselling intervention and control group participants experienced a similar reduction in CG symptoms at postmeasure. However, intervention participants demonstrated a greater reduction in symptom levels at follow‐up (M = 53.64; d = .33) compared to the control group (M = 62.00). Results suggest community‐based bereavement counselling may have long‐term beneficial effects. Further longitudinal treatment effect investigations with extensive study intervals are needed.

## 1 INTRODUCTION

Research and professional consensus suggest that the majority of bereaved people will cope with the pain of a “normal” grief reaction without professional help, and that, over time, they will begin to feel better (Kersting, Brähler, Glaesmer, & Wagner, 2011; Zisook et al., 2014). Still, bereavement is associated with a variety of negative mental and physical health outcomes (for reviews: Stroebe, Schut, & Stroebe, 2007; Zisook et al., 2014). Physical health outcomes include an increased risk of mortality (Buckley et al., 2012), suicidality (Prigerson et al., 1997; Zisook et al., 2014), and morbidity (Buckley et al., 2012; Keyes et al., 2014; O'Connor, Schultze‐Florey, Irwin, Arevalo, & Cole, 2014). Mental health outcomes include depression, post‐traumatic stress disorder (PTSD), and difficulties in grieving that extend in duration and severity beyond the scope of “normal” grief symptoms, also termed complicated grief (CG) (Zisook et al., 2014). For the sake of clarity, it is useful to note that varying labels and definitions of CG have been proposed (e.g., prolonged grief disorder, persistent complex bereavement disorder; American Psychiatric Association, 2013; Maercker et al., 2013; Prigerson et al., 2009; Shear, 2015), with estimates of the prevalence and incidence of CG varying according to the definition and the population observed (for a brief review, see Rosner, Pfoh, & Kotoučová, 2011).

Given the patterns of difference in reactions to a loss experience, it is critically important that effective psychological interventions are developed for people experiencing difficulties in coping with bereavement. Systematic reviews, including meta‐analyses, have identified the following characteristics of bereavement intervention programs that have been associated with intervention outcomes: (a) time since bereavement (interventions early after bereavement appear to be ineffective, cf. Bonanno, 2005; Currier, Neimeyer, & Berman, 2008; Schut, Stroebe, van den Bout, & Terheggen, 2001); (b) the way contact between client and counsellor is established (outreaching intervention being related to less positive effects than intervention instigated by the bereaved person; see Schut et al., 2001); and (c) the initial level of distress of the bereaved person and risk factors for experiencing complications grieving. Concerning the latter set of characteristics, it has been demonstrated that higher distress correlates with better results (cf. Prigerson & Jacobs, 2001; Shear, 2015; Zisook et al., 2014). Interventions aimed at treating bereaved people with high levels of grief‐related distress have been termed tertiary interventions and have been associated with positive outcomes (Schut et al., 2001). Outcomes for interventions focused on treating bereaved people with risk factors for developing complications, termed secondary interventions, have produced conflicting results (Schut et al., 2001). Nevertheless, recent investigations where participants were screened for risk factors have indicated better results for higher risk groups (see Burke & Neimeyer, 2013; Litz et al., 2014; Shear, 2015).

It can be surmised that an effective intervention would be aimed specifically at help‐seeking bereaved people at least 6 months after bereavement who present with, or are seriously at risk of developing, psychopathology (Center for the Advancement of Health, 2003; Currier et al., 2008; Schut et al., 2001; Wimpenny et al., 2007). In line with these findings, meta‐analysis showed that psychotherapeutic interventions yield moderate effect sizes in help‐seeking bereaved people with elevated levels of grief or CG more than 6 months after loss (Wittouck, Van Autreve, De Jaegere, Portzky, & van Heeringen, 2011; for examples of effective interventions: Boelen, de Keijser, van den Hout, & van den Bout, 2007; Shear & Shair, 2005).

Several recent randomized trials of psychotherapy for CG similarly yield moderate to strong effects when targeting indicated groups of bereaved people (e.g., Eisma et al., 2015; Litz et al., 2014; Rosner, Pfoh, Kotoučová, & Hagl, 2014; for a review: Doering & Eisma, 2016). Although these studies are of major importance in identifying which interventions work best for bereaved people experiencing complications in the grieving process, there are common limitations to many of them. First, due to practical and ethical limitations, most studies only assess short‐term effects, which makes it difficult to assess any longer term benefits (for notable exceptions: De Groot et al., 2007; Kersting et al., 2013; Rosner, Bartl, Pfoh, Kotoučová, & Hagl, 2015). Second, the majority of recent studies concern interventions that are offered in a professional setting and not in the setting in which they are most often delivered; in the U.K., the majority of bereavement intervention services are provided by non‐profit sector staff and volunteers (estimated at 70%–90% in the U.K. by Stephen et al., 2009, and similar situations of bereavement care offered by palliative care and hospice organizations in Australia, Japan, and the United States have been described by Breen, Aoun, O'Connor, & Rumbold, 2014).

### 1.1 The present study

A unique situation of bereavement care in Scotland and a newly developed intervention at a Scottish national organization, Cruse Bereavement Care Scotland (CBCS), have presented the opportunity to address these gaps in the research by conducting a naturalistic, longitudinal study on the effectiveness of community‐based, one‐to‐one bereavement counselling. The CBCS intervention entails an intake assessment process to ensure the provision of care for bereaved people at the appropriate time (i.e., more than 6 months after loss) on an in‐reaching basis. Pre‐existing logistical issues (described below in greater detail) also provided for a quasi‐randomized, no‐intervention control condition to balance the study design.

The development of the CBCS model of bereavement intervention was theory‐driven and guided by Schut and Stroebe's dual process model of coping with bereavement (Stroebe & Schut, 1999). Following this model, bereaved people who engage in an adaptive coping process oscillate between loss‐oriented and restoration‐oriented behaviours, which facilitate positive and negative reappraisals. Bereaved people who experience complications may require support in finding pathways to permit this oscillation in everyday life. To offer this kind of support, the CBCS model was developed as a flexible, bereavement‐specific counselling intervention, combining elements of a number of established therapeutic methods including cognitive behavioural therapy (Boelen et al., 2007), person‐centred counselling (Larson, 2013), and the psychodynamic approach (Mikulincer & Shaver, 2008). Details of the CBCS intervention model have been discussed in a qualitative study by Simonsen and Cooper (2015).

The aim of the present study was to establish—uniquely—whether grief counselling offered through a community‐based organization to help‐seeking, highly distressed, and/or high‐risk bereaved individuals is effective in improving psychological and social functioning relative to a no‐intervention control. There were three primary hypotheses. First, in line with the above‐mentioned meta‐analyses, we predicted a greater decline in CG symptom levels in the intervention as compared with the control group at post‐test (1 year after baseline). Second, following indications in the literature of a diminishment of effect with the passage of time (Currier et al., 2008), we expected to see a smaller difference between the slopes of symptom level change between the intervention and the control group in the postmeasure to follow‐up interval (one and a half years after baseline). Third, we also expected the difference within the study categories to be smaller between post‐test and follow‐up, given the potential for posttherapy relapse. Our secondary hypotheses predicted a similar pattern of results for symptoms associated with PTSD and symptoms of general psychological distress.

**Key Practitioner Messages**

- Bereavement counselling for elevated‐ and high‐risk bereaved persons has a beneficial effect on grief symptoms over 18 months.
- Preliminary indications suggest no marked difference in the effectiveness of bereavement counselling for elevated versus high levels of complicated grief.
- Professionally trained volunteer counselling by a non‐profit organization complements professional services.

## 2 METHOD

### 2.1 Procedure

The present study was approved by the NHS East of Scotland Research Ethics Committee (IRAS project ID 56758) in November 2010, with study progress reports submitted on an annual basis until 2015. Recruitment for the study took place between January and September 2011 across all mainland locations of CBCS, an independent, voluntary sector organization specialized in delivering informational and counselling support to bereaved people at no cost to them. Each year, approximately 12,000 people contact CBCS for information and support, and about 55,000 hr of service are provided to the Scottish community.

Participants of this study were adult (age 18+) residents across Scotland who had been bereaved for at least 6 months, had already received basic written information in the form of leaflets on coping with bereavement (CBCS, 2010; Kuykendall, 2005), and had contacted CBCS to request for one‐to‐one counselling support. Eligibility for the study was limited to people who were actively seeking help (i.e., no routine referrals) and were eligible to receive standard care from CBCS. Excluded from the study on these grounds were people with learning difficulties, cognitive difficulties, or special communication needs (because these clients are allocated directly to volunteers with specialized skills); people presenting with co‐morbid psychological conditions such as substance abuse problems, schizophrenia, or psychosis (to whom referrals for specialist care were provided); and people presenting with strong suicidal ideation (who were supported following a different protocol). People receiving external psychological support were also excluded from the study. Use of anti‐depressant and antianxiety medication among study participants at baseline was not an exclusion criterion if use of medication was consistent throughout data collection. This choice was made due to the widespread use of anti‐depressants and antianxiety medication in Scotland (The Scottish Government, 2014; cf. Shear & Shair, 2005).

Study information packs, including a written invitation to participate, were provided to eligible people either in person after a visit to CBCS or by post following telephone contact. Participants indicated their agreement to enrol in the study by signing and returning a study consent form by freepost. Approximately 1,400 packs were distributed, and 349 people agreed to participate (24.9%; this rate of acceptance is not unusual for bereavement research, as discussed below in Section 3.1). Of these, five people were excluded from the study (due to external professional help received), and 344 were enrolled in the study. Assignment to study conditions was quasi‐randomized. Quasi‐randomization enabled us to include a no‐intervention control group and a participant observation period of 18 months, while adhering to ethical standards, and not denying care to help‐seeking bereaved people. Participants for whom counselling sessions could be scheduled were assigned to the intervention condition (*n =* 156). The control group consisted of those who wanted counselling but were unable to participate (in the near future; *n* = 188). The majority of these were people who could not find a mutually agreeable time for counselling sessions and/or could not arrange transport to the service location. (Although CBCS has locations throughout Scotland, the topography of Scotland is such that a nearby location may in reality be difficult to reach.) There was also a small number of participants who remained on a waiting list for intervention services due to the organization's limited capacity (at that time) to provide care in the short term.

### 2.2 Sample characteristics

Demographic details and loss‐related characteristics of the study sample are presented in Table 1 (for group comparisons, see results). Seventy‐nine percent of participants were females. 1For further comments on this characteristic, please see below in Section 4. The mean age of participants was 49.3 years (*SD* = 14.20), with a range from 20 to 85 years. The majority of the sample had lost a partner (38%) or parent (37%) within the previous 2 years (80%) and reported that the death had been unexpected (63%).

**Table 1:** Baseline personal characteristics of control and Cruse Bereavement Care Scotland (CBCS) participants

	Control		CBCS	
	(*N* = 188)		(*N* = 156)	
Demographic characteristics				
Age in years (mean [*SD*])	48.5	(14.17)	50.1	(14.48)

	*n*	(valid %)	*n*	(valid %)
Gender	188		156	
Female	146	(77.66%)	125	(80.13%)
Income	150		137	
Below median	62	(41.33%)	40	(29.20%)
Median	34	(22.67%)	46	(33.58%)
Above median	54	(36.00%)	51	(37.23%)
Living with a partner	181		153	
Yes	52	(28.73%)	51	(33.33%)
No	129	(71.27%)	102	(66.67%)
Participant has children**	185		153	
No	53	(28.65%)	63	(41.18%)
Yes	132	(71.35%)	90	(58.82%)
Use of medication at baseline*	183		156	
No	91	(49.73%)	91	(58.33%)
Yes, antianxiety/anti‐depressants	84	(45.90%)	58	(37.18%)
Yes, other (incl. sleeping pills)	8	(4.37%)	7	(4.49%)
Loss‐related characteristics				
Time since the death*	180		154	
6 months	38	(21.11%)	37	(24.03%)
Between 6–12 months ago	64	(35.56%)	58	(37.66%)
1–2 years ago	44	(24.44%)	35	(22.73%)
2–5 years	23	(12.78%)	18	(11.69%)
5+ years ago	11	(6.11%)	6	(3.90%)
Relationship to the deceased**	186		155	
Partner	75	(40.32%)	54	(34.84%)
Parent	75	(40.32%)	53	(34.19%)
Sibling	11	(5.91%)	17	(10.97%)
Child	18	(9.68%)	19	(12.26%)
Other friend/relative	7	(3.76%)	12	(7.74%)
Quality of the relationship	184		155	
Very good/good	169	(91.85%)	144	(92.90%)
Reasonable	7	(3.80%)	7	(4.52%)
Not good/bad	8	(4.35%)	3	(1.94%)
No relations	0		1	(0.65%)
Expectedness of the death	182		154	
Expected	35	(19.23%)	33	(21.43%)
Neither expected nor unexpected	30	(16.48%)	22	(14.29%)
Unexpected	117	(64.29%)	99	(64.29%)
Other resources contacted (prior to study)**	182		153	
No	116	(63.74%)	123	(80.39%)
Yes (psychiatrist)	9	(4.95%)	5	(3.27%)
Yes (psychologist, counsellor, therapist)	38	(20.88%)	18	(11.76%)
Yes (support group, other social organization)	14	(7.69%)	7	(4.58%)
Outcome measures at T1 (*unadjusted means*)				

	*M*	*SD*	*M*	*SD*
Impact of Event Scale	44.96	19.68	43.04	18.19
Inventory of Complicated Grief—Revised	68.42	25.86	64.78	23.68
Symptom Checklist 90—R	151.72	80.10	132.33	71.11

* *p* < .05.

** *p* < .001.

### 2.3 Intervention

The CBCS intervention model was developed specifically for the provision of one‐to‐one counselling support to adults who are experiencing difficulties in coping with bereavement. Informed by the dual process model of coping with bereavement (Stroebe & Schut, 1999), the intervention aims in part to normalize the participant's grief reaction when needed and to create a safe holding environment for the participant to express and confront emotions relating to the bereavement. This pluralistic model of bereavement intervention incorporates components of three therapeutic traditions. From the person‐centred approach, these include unconditional positive regard, empathy, and congruence; from CBT, they include guided exposure exercises adapted to meet individual needs; and from the psychodynamic tradition, they include an awareness of attachment patterns that may inform grief reactions. (For more detail, as well as a client's perspective, see Simonsen & Cooper, 2015.)

At baseline, an intake assessment was conducted by a purpose‐trained volunteer using the IBACS, a validated assessment instrument for bereavement‐related grief and risk factors (Newsom, Schut, Stroebe, Birrell, & Wilson, 2016; Newsom, Schut, Stroebe, Wilson, & Birrell, 2016). The minimum IBACS score was 0; the maximum was 55. Clinical indications for the IBACS currently maintained a minimum threshold of 19 points for bereavement intervention. Bereaved people who received fewer than 19 points were considered to be coping effectively on their own, and intervention is not indicated. Likewise, people who received more than 54 points were to be considered in need of urgent specialist care and would be accordingly referred to qualified professional resources. For bereaved people who received from 19 to 54 IBACS points, two further subcategorizations facilitated the assignment of appropriately experienced counsellors. Participants who received an IBACS score between 19 and 28, which corresponds with marginal levels of CG, were allocated to the skilled listener category. Placing the current intervention design in the context of existing categories (following the indications from Schut et al., 2001) described in the introduction, counselling for this category would be considered a *secondary* intervention, because it was a preventative intervention for people with elevated symptoms of grief who were at risk of developing CG. Participants who reached an IBACS score of 29 to 54, indicating high CG levels, were allocated to the advanced skills/counsellor category. This score range roughly corresponds with CG caseness, and counselling in this category would be considered a *tertiary* intervention in Schut et al.'s (2001) framework.

Counsellors working with participants in the skilled listener category and those working with participants in the advanced skills listener category offer support based on the same intervention model; only their hours of experience are different. All counsellors have completed (at a minimum) a year‐long diploma‐level course in counselling (certified by the Scottish counselling authority, COSCA) as well as a COSCA‐certified grief‐specific training module (details can be requested through the national office of CBCS). Counsellors are promoted from the skilled listener to the advanced skills counsellor category after completing an additional 60 hr of supervised, client‐facing work, along with requisite continuing professional development hours. Sixty‐six volunteer counsellors delivered care in this study. Participants worked with the same counsellor at each session. Counselling sessions were held at CBCS locations across Scotland in quiet rooms with comfortable, non‐clinical furnishings. Participants attended counselling sessions on a weekly basis, scheduled at their convenience in the day or evening. Sessions lasted 50 min. Standard CBCS procedure is to offer clients six sessions, though a participant and counsellor may mutually agree to adjust the number of sessions as needed. An average of six sessions of counselling was offered (*M =* 6.32, *SD =* 3.09).

### 2.4 Data collection procedure

Data for the present study were collected at three time points: baseline, T1; posttreatment, T2 (T1 + 12 months); and follow‐up, T3 (T1 + 18 months). Time intervals were standardized to be uniform across conditions. Duration of the period from intake through completion of intervention varied from participant to participant, with an average length of 9 months. Because the intake plus intervention period was universally completed by 12 months, we selected 12 months as a standard interval between T1 and T2.

### 2.5 Measures

Five instruments were used in the present investigation: a measure of demographic and loss‐related characteristics (administered at T1); an intake assessment instrument measuring bereavement‐related distress and risk of developing complications (administered at T1); a primary measure for CG symptoms; and secondary measures to assess PTSD symptoms and general psychological health symptoms (administered at T1, T2, and T3).

#### 2.5.1 Bereavement‐related distress and risk of complication (intake assessment)

IBACS (Newsom, Schut, Stroebe, Wilson, et al., 2016) was administered to measure baseline levels of bereavement‐related grief symptoms and the risk of developing complications while grieving due to the presence of known risk factors. Risk factors were assessed through a semistructured interview; symptoms of bereavement‐related distress were measured with a structured question set. A validation exercise for the IBACS demonstrated convergent validity with other instruments including the Inventory of Complicated Grief—Revised (ICG‐R; Prigerson & Jacobs, 2001; *r* = .82, *p* < .01) and cut‐off score convergence with CG “caseness” calculations using the ICG‐R (Newsom, Schut, Stroebe, Birrell, et al., 2016).

#### 2.5.2 Demographic and loss‐related characteristics

A self‐constructed questionnaire was used to assess demographic characteristics (gender, age, lives with a partner (yes/no), has children (yes/no), and income) and loss‐related characteristics (time since loss; relationship with the deceased; expectedness of the death; quality of the relationship with the deceased; and medication use).

#### 2.5.3 Complicated grief

Symptoms of CG were assessed with the 30‐item ICG‐R (Prigerson & Jacobs, 2001). Strong internal consistency (Cronbach's α = .94) has been reported for the ICG‐R (Prigerson & Jacobs, 2001). Convergent validity for the ICG (Dutch version) was demonstrated through a high correlation (*r* = .71, *p* < .05) with The Texas Revised Inventory of Grief (Boelen, Van den Bout, De Keijser, & Hoijtink, 2003). Convergence with the outcome of the structured clinical interview protocol, the Traumatic Grief Evaluation of Response to Loss (Prigerson & Jacobs, 2001) confirmed construct validity. In the present investigation, baseline reliability was excellent, α = .95, and was minimally higher at the two subsequent time points (α = .96).

#### 2.5.4 PTSD‐related symptoms

Symptoms of avoidance, hyper‐arousal, and intrusion were assessed using the Impact of Event Scale—Revised (IES‐R). The IES‐R is a 22‐item questionnaire, addressing the above‐listed symptoms, which conform to DSM‐IV criteria for PTSD. Strong test–retest reliability (*r* = .89 to .94) has been reported for the IES‐R (Weiss & Marmar, 1996), and convergent validity with the PTSD checklist has been demonstrated with a high correlation of *r* = .84 (Creamer, Bell, & Failla, 2003). In the present study, reliability was excellent at baseline, α = .92, and continued to be at T2, α = .94, and at T3, α = .95.

#### 2.5.5 General psychological symptoms

The Symptom Checklist 90 Revised (SCL‐90‐R; Derogatis & Cleary, 1977) is a 90‐item questionnaire addressing general psychological health symptoms that can be divided into nine domains: anxiety, depression, hostility, interpersonal sensitivity, obsessive compulsive symptoms, paranoid ideation, phobic anxiety, psychoticism, and somatization. Internal consistency has been shown to range from α = .74 to .97 (Prinz et al., 2013). Each domain has demonstrated construct validity (Derogatis & Cleary, 1977), and validity as a unidimensional scale has been established for the depression, phobic anxiety, and interpersonal sensitivity subscales (Bech, Bille, Møller, Hellström, & Østergaard, 2014). Average positive symptom scores can be used to calculate the Global Severity Index, a general symptom severity rating, which was used in the present study (Derogatis & Cleary, 1977; Derogatis & Unger, 2010). Baseline reliability was excellent in the present investigation, α = .98, and maintained at both T2 and T3.

### 2.6 Statistical analyses

Three main operational hypotheses were formulated to investigate the primary research question. We expected to find (a) the slope (i.e., decline) of mean grief symptoms in the intervention condition would be greater than in the control condition between T1 and T2; (b) a difference between the slopes of change of the intervention and the control conditions between T2 and T3; (c) a greater slope of change between T1 and T2 than between T2 and T3 in both study categories (this hypothesis was tested separately for each group). Next, the same hypotheses were to be tested for secondary outcomes (i.e., PTSD and general psychological health symptoms).

A random effects model for fixed occasions was developed to analyse the data (Snijders & Bosker, 2012). An average score was estimated for the outcome variable at each time point for the intervention and control conditions. Where baseline differences in demographic or loss‐related variables between conditions were detected, these variables were added as covariates. Assumptions of linearity, multicollinearity, homoscedasticity, and normality of residuals were tested prior to analysis. To account for the longitudinal nature of the data, a multilevel regression was conducted using MLwiN (Rasbash, Steele, Browne, & Goldstein, 2015), with repeated measures nested within subjects, and a random effect of time included to account for within‐subject correlations. Estimation of model parameters and standard errors was conducted with full information maximum likelihood with robust standard errors.

Change in outcome variables across time was measured by calculating the slope of change of adjusted mean scores for both study conditions. For each condition, the adjusted mean at T2 was subtracted from that of T1, and the adjusted mean at T3 from that at T2. Positive values indicate a decline of grief‐related symptom scores between time points. The slopes were compared across study conditions to answer research questions 1 and 2 and across time within both study conditions separately to answer research question 3. Two‐sided tests were conducted with a significance level of α = 0.05.

Following Wang et al. (2015), a stepwise serial gatekeeping procedure was selected to address multiple hypotheses testing in the output of the model and reduce the risk of Type 1 (false positive) errors. This procedure affords the flexibility of testing families of hypotheses in order of their relevance to the main research question, reserving power for the most critical research questions.

The first step of the process was to establish a hierarchy in the families of hypotheses. Because we planned to test our three operational hypotheses on three different sets of outcome measures (i.e., ICG‐R, IES‐R, and SCL‐90‐R), we structured the analysis by placing the three operational hypotheses into three families, each defined according to the relevance of the outcome measures to the objectives of the bereavement intervention. Because symptoms of CG (ICG‐R) are bereavement‐specific complaints, this was the primary outcome measure, and its hypotheses were the first family in the hierarchy. PTSD symptomatology (IES‐R) was a secondary outcome measure and the second family of hypotheses. This was due to the close association between the instruments' subscale measures (avoidance, intrusion, and hyper‐arousal) with grief symptoms (e.g., Boelen & Eisma, 2015; Boelen, Huntjens, van Deursen, & van den Hout, 2010). The family of hypotheses concerning general symptoms (SCL‐90‐R) was placed third, because the impact of bereavement intervention on these nongrief‐specific measures was less direct.

Significance testing was then conducted, with the four null hypotheses for the primary outcome variable, grief, tested at α = 0.05. Only if all of these were rejected would the null hypotheses for the secondary outcomes be tested and so forth down the hierarchy of families. In other words, if any null hypotheses in the family were not rejected, results of this first family of analysis would be retained, and no testing of subsequent families would be conducted.

Normality of residuals at both the repeated measures level and subject level was checked by means of Quantile‐Quintile (QQ) plots. Homoscedasticity of the residuals at both levels was checked by means of scatter plots in which the residuals were plotted as a function of predicted scores.

### 2.7 Clinical significance

To evaluate the clinical significance of results within the framework of a naturalistic study where absolute recovery is not expected but a demonstrated reduction in symptoms is meaningful (cf. Wise, 2004), a categorical CG caseness variable was calculated by following the indications by Prigerson and Jacobs (2001) using a specific selection of items from the ICG (cf. Boelen et al., 2007). The variable was used to indicate whether mean CG symptoms exceeded a minimum clinical threshold value. To compare CG caseness across conditions, a crosstabs analysis was conducted in a subset of the data that only included participants who had provided complete data sets at all three measurement points.

Lastly, based on the ambiguous indications in the literature concerning the effectiveness and clinical relevance of secondary interventions, the opportunity was taken to conduct an additional exploratory analysis to determine whether there were differences in symptom changes between participants (within the intervention condition) who received intervention on a secondary or preventative basis and those who received it on a tertiary or indicated basis. Using a mixed, repeated measures analysis of variance and omitting incomplete cases, we compared the magnitude of symptom change among participants who received intervention on a secondary basis and those who received it on a tertiary basis across time, specifically the intervals between T1 and T2; T2 and T3; and T1 and T3. Although this test could provide only preliminary information, we believed this exploratory step would provide useful indications for future research.

## 3 RESULTS

### 3.1 Participant flow

**Figure 1:**
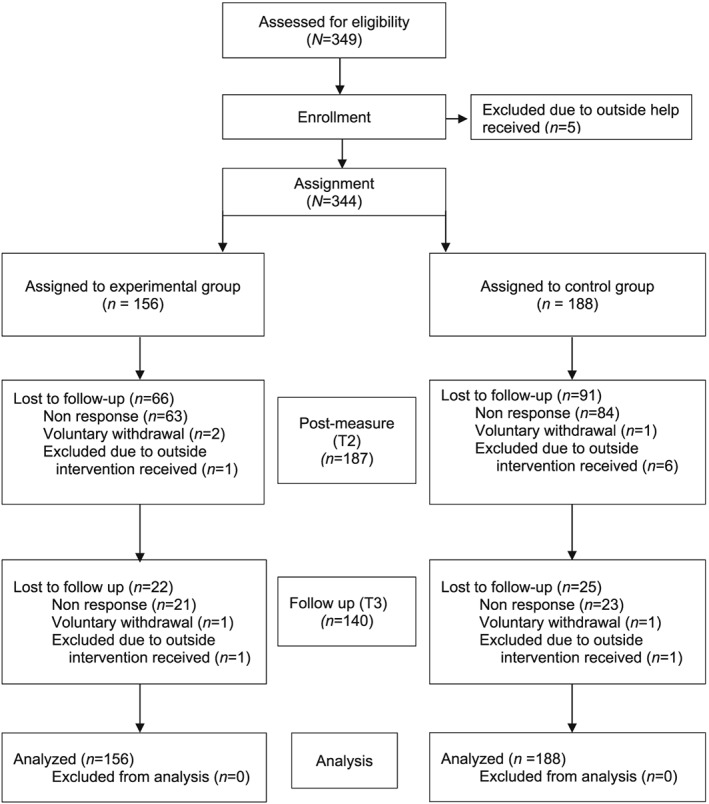
Flow of participants

Figure 1 provides details of study attrition by condition across time points. The response rate for all three time points (cumulatively) was 41%. Though the attrition rate may appear high, it is not unusual for longitudinal bereavement research involving postal questionnaires (Aoun et al., 2015). As Figure 1 illustrates, the majority of study attrition was through nonresponse. In order to respect participants' decisions to withdraw and to protect their confidentiality, after one reminder letter, no further steps were taken to track nonresponsive participants. Voluntary withdrawal from the study (*n =* 5) was indicated either by telephone or through written notification by post. Study withdrawal was attributed to no longer wishing to reflect on their bereavement experiences. Of these, three participants attributed their decisions to feeling better, and two stated that reflecting on the bereavement evoked unwanted sad feelings. Nine participants were excluded because they received outside professional support for bereavement.

### 3.2 Attrition

Completers of all measurements and noncompleters did not significantly differ on gender, use of medication, expectedness of the death, contact with other support services prior to CBCS, or levels of CG, post‐traumatic stress, and general psychological functioning. However, a medium‐sized difference (*d* = .32) was detected for age (study completers were older at baseline [*M* = 52.23, *SD* = 13.14] than noncompleters [*M* = 47.86, *SD* = 13.80; *t*(336) = 2.91, *p* = .004]). Small differences were also detected for kinship (those who lost a partner were less likely to drop out; those who lost a parent were more likely to drop out, χ^2^(4) = 15.14, *p* = .004, *r* = .21) and time since bereavement (more participants who had been bereaved just 6 months prior to baseline were more likely to drop out; participants who were bereaved 1 to 2 years prior to baseline were less likely to drop out, χ^2^(4) = 16.78, *p* = .002, *r* = .22).

Completers also had a lower mean IES‐R scores at baseline (*M* = 41.62, *SD* = 18.49) than noncompleters, *M* = 45.81, *SD* = 19.23; *t*(338) = 2.10, *p* = .045, though again this difference was small, *d* = .22.

### 3.3 Baseline characteristics

Table 1 shows baseline measures of demographic and loss‐related characteristics, and outcome measure means of the study sample by condition. Chi‐square tests revealed no significant differences between participants in the intervention and control conditions with respect to gender, household income, expectedness of the death, the reported quality of the relationship to the deceased person, or whether the participant was living with a partner*.* The *t* tests indicated that age and outcome measure scores were also not significantly different across conditions.

Nevertheless, some differences between groups existed at baseline. Control group participants were more likely to have lost a partner or parent, χ^2^(5) = 22.39, *p* < .001; contacted other support resources prior to the study, χ^2^(6) = 31.68, *p* < .001; and used antianxiety or anti‐depression medication at baseline, χ^2^(2) = 8.08, *p* = .018. There was also a slight difference in the time since the death, detected in an independent sample *t* test treating time since loss as an ordinal variable, *t*(1000) = 2.02, *p =* .044. These four variables (kinship, resources contacted, medication use, and time since the death) were used as covariates in the main analyses.

### 3.4 Statistical tests

The overall focus of our analysis was the comparison of two slopes across conditions (intervention vs. control) for our three outcome measures, CG, post‐traumatic stress, and general psychological health. We compared two slopes: slope 1 (between T1 and T2) and slope 2 (between T2 and T3). As noted, we expected that there would be differences between the slopes across conditions (Hypotheses 1 and 2). We also expected that in both study conditions, the first slope would differ from the second (Hypothesis 3).

### 3.5 Model assumptions for a linear multilevel model

The outcomes in the present analysis were sum scores of subscales with a large enough number of values for the variables to be treated as continuous variables. Scatterplots were generated to examine the linearity of the relationship between sum score outcomes and the continuous variable covariate, time since death, which was an ordinal variable with eight categories. Scatterplots indicated a linear relationship between outcome and time since the death. The variance inflation factor (VIF) was calculated using dummy variables for each nominal variable, and the results (VIF < 10) ruled out multicollinearity. An examination of scatterplots of residuals at both levels revealed no violation of the assumption of homoscedasticity, and PP plots showed no violation of normal distribution of residuals. Histograms indicated a normal distribution of variables at T1 for the ICG and the IES‐R sum score. SCL‐90 sum scores were not normally distributed; however, this was not unexpected due to the inclusion criteria of the study, which limited participation to those people who presented with elevated levels of grief symptoms. Although histogram plotting revealed that the sum scores did not all follow a normal distribution, this did not present a problem because the multilevel model assumes a normal distribution of residuals.

### 3.6 Complicated grief symptoms (ICG)

**Figure 2:**
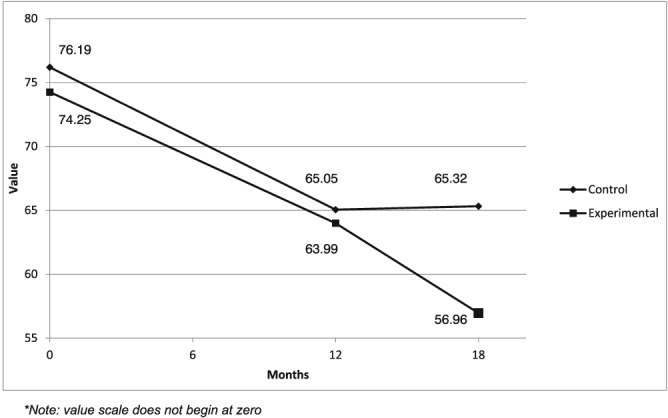
Inventory of Complicated Grief mean scores (adjusted for kinship to the deceased person, resources contacted, medication use, and time since the death) across time points

Figure 2 illustrates the covariate‐adjusted means of the ICG scores across conditions over the three time points. The first family of tests contained the three groupings of hypotheses for CG symptoms measured by the ICG. Results for Hypothesis 1 indicated that symptom levels of CG declined over the 12 months between T1 and T2 in both study conditions with a similar slope of change, χ^2^(1) = .113, *p* = .727. In the intervention condition, the mean of grief symptom levels decreased from T1 (*M* = 70.93) to T2 (*M* = 60.68), and in the control condition, the mean decreased from T1 (*M* = 72.87) to T2 (*M* = 61.73). The difference in the decrease in grief levels between the two study conditions at these time points was not statistically significant. This indicates a rejection of Hypothesis 1, as the intervention had no apparent impact at the post‐test shortly after its completion.

For Hypothesis 2, it was postulated that the slope of symptoms would differ between the intervention group and the control category between T2 and T3. Results confirmed Hypothesis 2 and revealed that the change in mean CG scores over the 6 months between T2 and T3 was greater for the intervention condition, with a decline from T2 (*M* = 60.68) to T3 (*M =* 53.64), compared to the control group, where mean symptom scores increased from T2 (*M* = 61.73) to T3 (*M =* 62.00), χ^2^(1) = 6.01, *p* = .014, *d =* .33. Complicated grief symptoms decreased relatively more in the intervention group between postmeasure and follow‐up—after bereavement, counselling was completed for some time—than between baseline and postmeasure, when participants had just completed counselling.

Tests with the ICG also confirmed Hypothesis 3, which suggested that the slope of change in symptom levels would be greater within the intervention category and the control category between T1 and T2 than between T2 and T3. Results indicated that, for both groups, the slope of within‐group change of mean symptom levels was greater between T1 and T2 than between T2 and T3, χ^2^(1) = 5.48, *p* = .019. Within the intervention group, the mean decreased 7.03 points between T2 and T3 compared to a decrease between T1 and T2 of 10.3 points. The effect size of this difference was small (*d =* .27). Within the control group, the mean decrease between T2 and T3 was negligible (0.27 points). The mean decrease between T1 and T2, in contrast, was 11.0 points, with a small‐to‐medium effect size (*d =* .43).

Although *p* values for the results of Hypotheses 2 and 3 for CG were sufficient to reject the null hypotheses, the *p* value for Hypothesis 1 was not. Following the serial gatekeeping procedure, to reduce the possibility of a false positive, results for this family were retained, and no further testing of families in the dataset was conducted. All null hypotheses for the second group of hypotheses, and indeed all subsequent groups, were therefore not tested.

### 3.7 Change criteria

**Figure 3:**
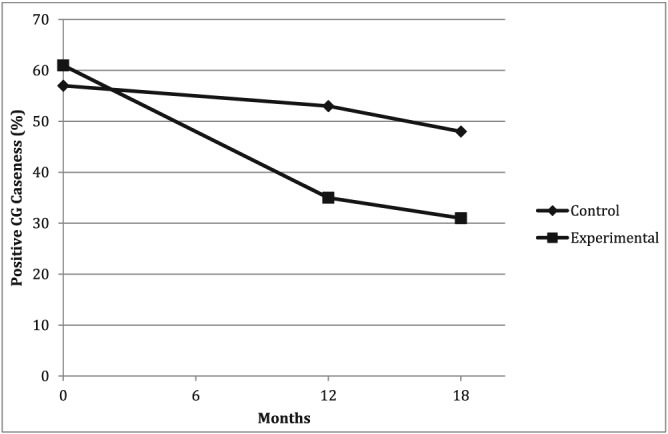
Change in complicated grief (CG) caseness over time, indicating above‐threshold level symptoms of CG measured using the Inventory of Complicated Grief

Figure 3 illustrates changes in CG caseness per study condition across the three time points. As an indicator of clinically meaningful change, CG caseness was calculated in both study conditions across time points and compared in a crosstabs analysis. Only cases with complete data across all three measurement points were included in the analysis; therefore, the available sample was smaller (*n =* 116; 62 intervention, 54 control) than the sample for the multilevel model. At baseline (T1), positive CG caseness was detected in 61% (*n =* 38) of the intervention conditions (*M =* .61, *SD =* .49) and 57% (*n* = 31) of the control conditions (*M =* .57, *SD =* .50), with no notable difference between the groups; *t*(114) = .422, *p =* .674. At follow‐up (T3), CG caseness was reduced to 31% (*n* = 19) of the intervention condition (*M =* .31, *SD =* .46) compared to 48% (*n =* 26) of the control group (*M =* .48, *SD =* .50). The difference between the groups at T3 indicated only marginal statistical significance, *t*(114) = 1.94, *p =* .054.

Using the intervention category participants from this same subset of the sample, a mixed repeated measures analysis of variance was conducted to explore the differences in the change in grief symptom levels for secondary and tertiary intervention category participants over time. The sample was again restricted to intervention category participants who had complete data sets at all three time points (the inclusion of item‐level T2 data in this analysis made the sample slightly smaller, *n =* 56; secondary *n* = 31; tertiary *n* = 25). The difference in symptom levels on the ICG was calculated as a continuous variable for T1–T2, T2–T3, and T1–T3 intervals. No interaction was detected between the intervention basis (secondary or tertiary) and time—Wilks' lambda = .96, *F*(2, 53) = 1.04, *p* = . 359, partial eta squared = .038—indicated a small to moderate effect size. A substantial main effect for time was detected—Wilks' lambda = .59, *F*(2, 53) = 18.61, *p* = <.001, partial eta squared = .413—denoting a large effect size and indicating that both secondary and tertiary intervention categories experienced a decrease in symptom levels across time. The main effect comparing secondary and tertiary interventions indicated no detectable differences, *F*(1, 54) = .517, *p* = .475, partial eta squared = .009. The results suggest that there may be no difference in the change in CG symptom levels when bereavement intervention is offered on a secondary or a tertiary basis.

## 4 DISCUSSION

The objective of this controlled intervention study was to test the effectiveness of a widely available approach to bereavement counselling in reducing symptoms of grief‐related distress among help‐seeking, bereaved adults. Like most bereavement intervention, as noted in our introduction, the intervention was offered through the voluntary (non‐profit) sector. Results of the investigation indicated that people who received bereavement counselling experienced a greater decrease in grief symptoms over time than a bereaved cohort that did not receive counselling. Time itself was found to have a strong effect on levels of grief in both conditions between baseline and 12‐month follow‐up. In the intervention group, grief symptom levels continued to decline; however, between the 12‐month postmeasure and the 18‐month follow‐up—*after counselling had been completed*—the control group's mean symptom levels remained unchanged. It therefore appears that distressed and at‐risk bereaved people who receive counselling experience a reduction in CG symptoms in addition to the effect of time after the counselling is completed. Due to corrections for multiple hypothesis testing, we were unable to produce results for the other outcome variables included in the analysis. Though it appeared that no, or at best minimal, effects would be detected for bereavement counselling on symptoms of PTSD or general psychological health, further research is needed to make a valid assessment.

When considering the clinical implications of these results, two observations are notable. First, more clinical improvement was observed in CG symptom levels (as indicated by an analysis of a grief caseness variable) among participants who had received counselling intervention; however, this effect was only marginally significant. Second, although the results of the investigation indicated that counselling intervention had a positive effect, this effect was markedly protracted. A primary factor contributing to this result was the reduction in symptom levels in the control condition that approximately matched that of the intervention condition between baseline and 12‐month follow‐up. Were it not for the second factor contributing to the study's results—the substantial drop in CG symptoms *after* counselling in the intervention group relative to the control group—we might have considered the intervention to have had no effect at all.

One explanation for this delayed effect may be the experience of grief counselling itself. Because counselling sessions require participants to confront difficult aspects of bereavement, it is possible that intervention participants experienced slightly elevated grief symptom levels during the counselling period and even the short term thereafter, leading up to postmeasure. Then, in the months following the post‐test, intervention participants' grief symptoms once again declined, and ultimately due to counselling, this decline was steeper than in the control group. In other words, the effort of “working through,” which is integral to counselling, would initially be reflected in elevated grief scores, then by a decline once these disturbing aspects were dealt with.

This specific effect has not been demonstrated in previous psychotherapy research for CG (e.g., Boelen et al., 2007; Rosner et al., 2014), so it is important to consider additional reasons for the delayed effect on CG. Differences in study design between the present and previous investigations could offer a partial explanation. Most notably, although the length of intervention in the present study varied, there was a full year between baseline and post‐test, which is longer than study intervals in nearly all previous intervention trials on CG employing a waitlist control (Doering & Eisma, 2016). It is therefore also possible that the healing effect of time may be more pronounced in our study compared to other trials.

The fact that greater symptom change was observed in the intervention group at 18‐month follow‐up is important for a number of reasons. First, for bereaved people who receive counselling, and for counsellors who provide it, the results provide a positive indication that counselling helps more than the passage of time alone. It may be that people initially attribute feeling better to counselling, whereas time alone might have reduced their suffering from grief as much; but in the long term, counselling has been shown to have a more beneficial effect and will reduce grief‐related symptoms further. For bereavement researchers, the results demonstrate the importance of a longitudinal study design, including a lengthy period between measurements. Data collection at additional time points both during and after counselling could also provide more insight into when changes in grief levels occur over time.

The effectiveness of community‐based counselling also suggests it to be a potentially cost‐effective alternative to professional grief counselling. Though the effect is modest, it must be noted that community‐based counselling initiatives currently have a larger reach than professional grief counselling interventions. Future research should include cost‐effectiveness analyses of different intervention alternatives for distressed bereaved persons.

### 4.1 Limitations

The present study's naturalistic, longitudinal design was selected for scientific as well as ethical reasons, but it also introduced limitations that should be considered when interpreting the results. First, our study sample was divided into conditions through a quasi‐randomized, and not a strictly random, procedure, which would have been preferable from a statistical perspective. Although previous meta‐analytic studies of psychotherapeutic interventions for bereavement showed no statistically significant differences between outcomes of studies with a randomized or a nonrandomized design (Currier et al., 2008), and despite our every effort to identify and control for any differences between our participant groups, it is impossible to be certain that there were no underlying differences that escaped measurement. A second limitation concerns the generalizability of results. Our sample, which was representative of the counselling organization's usual client base, was predominantly female, as is commonly the case in bereavement research and indeed in grief counselling at large (Stroebe, 2001). In addition, a majority of our sample was bereaved of a parent or partner. Though research has indicated that both the relationship to the deceased person and being a woman increased the likelihood of developing a CG reaction (Burke & Neimeyer, 2013), men have also been shown to be at greater risk of experiencing difficulties specifically after a spousal bereavement (Stroebe, 1998). Further investigation of the effectiveness of bereavement counselling for men and with greater differentiation among bereaved people's kinship ties to the deceased would be warranted. Lastly, excluding participants who take anti‐depressant or antianxiety medication would have been preferable; however, this was not feasible, given the indications that one in seven Scottish residents takes anti‐depressants (cf. The Scottish Government, 2014; BBC News, 2012). No differences were found in the use of these types of medications across study groups, however, and though it is difficult to assess whether prescription drugs had an impact on participants' CG symptoms, indications from a recent large‐scale trial by Shear et al. (2016) demonstrating a lack of efficacy for anti‐depressant medication alone on CG symptoms make it unlikely that medication use would have influenced outcomes. Lastly, though attrition analysis indicated some, predominantly small differences between completers and noncompleters, which were in line with participation patterns in bereavement research (Stroebe & Stroebe, 1990), it is worth noting that completers had lower PTSD symptoms at baseline. It is possible that people experiencing elevated PTSD‐related symptoms were underrepresented at posttreatment assessment, though the effect size for this difference was small.

### 4.2 Conclusion

The present study demonstrated the delayed, and possibly prolonged effect (at least for a period beyond a year) of a community‐based, pluralistic model of bereavement counselling for people at risk of developing, or already experiencing, CG. The investigation incorporated a number of recommendations from scientific research: waiting a number of months post bereavement before providing counselling; offering counselling on an in‐reaching basis; incorporating an intake assessment process to screen for CG symptoms and risk factors; and offering a tailored model of counselling. The effectiveness of this approach provides encouragement for supporting community‐based initiatives to promote psychological well‐being. Similar to an estimated 80%–90% of all bereavement support services in the U.K. (Stephen et al., 2009), the counselling service in this investigation was delivered by trained, professional‐grade volunteers at a non‐profit organization, where services are available to clients at no cost to themselves. Taken in a broader perspective, considering the long‐term effects of elevated grief symptoms (e.g., increased days in hospital) and costs of acute care (cf. Stephen et al., 2015), there are good reasons to conclude that community‐based bereavement counselling may allow health boards to increase the availability of support, reduce costs, and save rather than spend.
